# Approaches to improving mental healthcare for autistic people: systematic review

**DOI:** 10.1192/bjo.2024.707

**Published:** 2024-08-01

**Authors:** Sofia Loizou, Tamara Pemovska, Theodora Stefanidou, Una Foye, Ruth Cooper, Ariana Kular, Anna Greenburgh, Helen Baldwin, Jessica Griffiths, Katherine R. K. Saunders, Phoebe Barnett, Matilda Minchin, Gráinne Brady, Nafiso Ahmed, Jennie Parker, Beverley Chipp, Rachel R. Olive, Robin Jackson, Amanda Timmerman, Suzi Sapiets, Eva Driskell, Bethany Parsons, Debbie Spain, Vaso Totsika, Will Mandy, Richard Pender, Philippa Clery, Kylee Trevillion, Brynmor Lloyd-Evans, Alan Simpson, Sonia Johnson

**Affiliations:** National Institute for Health and Care Research (NIHR) Mental Health Policy Research Unit, King's College London, UK; NIHR Mental Health Policy Research Unit, Division of Psychiatry, University College London, UK; NIHR Mental Health Policy Research Unit, Division of Psychiatry, University College London, UK; Centre for Outcomes Research and Effectiveness, Research Department of Clinical, Educational and Health Psychology, University College London, UK; and National Collaborating Centre for Mental Health, Royal College of Psychiatrists, London, UK; Division of Psychiatry, University College London, UK; NIHR Mental Health Policy Research Unit Lived Experience Working Group, Division of Psychiatry, University College London, UK; and School of Health and Psychological Sciences, City, University of London, UK; NIHR Mental Health Policy Research Unit Lived Experience Working Group, Division of Psychiatry, University College London, UK; Lancaster and Morecambe Child and Adolescent Mental Health Services, Lancashire and South Cumbria NHS Foundation Trust, Morecambe, UK; and University of Wolverhampton, UK; Department of Clinical, Educational and Health Psychology, University College London, UK; Tizard Centre, University of Kent, UK; NHS England, London, UK; Division of Psychiatry, University College London, UK; and Camden and Islington NHS Foundation Trust, London, UK; NIHR Mental Health Policy Research Unit, Division of Psychiatry, University College London, UK; and Camden and Islington NHS Foundation Trust, London, UK

**Keywords:** Autism spectrum condition, mental health, adaptations, adults, mental healthcare

## Abstract

**Background:**

Autistic people have a high likelihood of developing mental health difficulties but a low chance of receiving effective mental healthcare. Therefore, there is a need to identify and examine strategies to improve mental healthcare for autistic people.

**Aims:**

To identify strategies that have been implemented to improve access, experiences of care and mental health outcomes for autistic adults, and to examine evidence on their acceptability, feasibility and effectiveness.

**Method:**

A co-produced systematic review was conducted. MEDLINE, PsycINFO, CINHAL, medRxiv and PsyArXiv were searched. We included all study designs reporting acceptability or feasibility outcomes and empirical quantitative study designs reporting effectiveness outcomes. Data were synthesised using a narrative approach.

**Results:**

A total of 30 articles were identified. These included 16 studies of adapted mental health interventions, eight studies of service improvements and six studies of bespoke mental health interventions developed for autistic people. There was no conclusive evidence on effectiveness. However, most bespoke and adapted approaches appeared to be feasible and acceptable. Identified adaptations appeared to be acceptable and feasible, including increasing knowledge and detection of autism, providing environmental adjustments and communication accommodations, accommodating individual differences and modifying the structure and content of interventions.

**Conclusion:**

Many identified strategies are feasible and acceptable, and can be readily implemented in services with the potential to make mental healthcare more suitable for autistic people, but important research gaps remain. Future research should address these and investigate a co-produced package of service improvement measures.

Autistic^‡^ people^1^ experience a high rate of mental health difficulties but are less likely to receive effective mental health support.^2^ For autistic people, co-occurring mental health difficulties can lead to negative outcomes including poor quality of life^3–5^ and increased risk of suicide.^6–8^ Accessing appropriate support from mental health services can be a critical step towards addressing mental health difficulties in autistic people. Dissatisfaction with care,^9^ high levels of unmet needs,^10^ and harmful effects^11^ suggest that mental health services do not currently provide sufficient support for many autistic individuals with co-occurring mental health difficulties. This can erode trust in services and prevent help-seeking in the future.^11^

Clinicians may struggle to distinguish autistic traits from symptoms of mental health conditions because of similarities in external presentation,^8,12–14^ which together with lack of clinician knowledge of autism^15^ creates difficulties for autistic people accessing and receiving appropriate mental health support. This may lead to delayed or missed autism diagnosis, misdiagnosis and ultimately ineffective treatment and support.^16^ Hence, there is a need to identify strategies to facilitate the detection of autism within mental health services.

Another potential barrier to obtaining high quality and appropriate mental health support is the lack of tailored care, as autistic people and their families recurrently report that services and treatment approaches are rarely adapted to their needs.^15^ Impediments to delivering appropriate support include lack of staff training and knowledge on how services could be adapted, and lack of evidence on best approaches.^11,17–20^ This scarcity of tailored approaches and lack of information about effective tailored care further highlights the need to identify strategies used to improve access, experiences of care and mental health outcomes for autistic people.

The aim of this review was to lay foundations for care that better meets the needs of autistic people, by identifying and examining strategies that intend to improve mental health treatment and care for autistic adults. This was addressed through the following research questions:

1. What strategies, including bespoke and adapted mental health interventions, service adaptations and strategies to detect autism, have been developed to improve mental healthcare for autistic people?
2. What is the acceptability and feasibility of strategies to improve mental healthcare for autistic people?
3. What is the effectiveness of strategies to improve mental healthcare for autistic people?

## Method

Our systematic review was conducted in accordance with the Preferred Reporting Items for Systematic Reviews and Meta-analyses (PRISMA) guidelines.^21^ A PRISMA checklist for this review can be found in Supplementary Table 1 available at https://doi.org/10.1192/bjo.2024.707. The review was commissioned by the National Institute for Health and Care Research Mental Health Policy Research Unit (a research unit funded to deliver evidence to inform mental health policy in England) to meet an identified need for more evidence to guide policy in this area. We developed a review protocol that was prospectively registered on PROSPERO (CRD42022347690). The review protocol was developed through consultations with the review's working group, which included lived experience researchers (i.e. people drawing on relevant lived experience to inform research), academics, clinicians and policy experts. All members of the working group had personal or professional expertise in autism and/or systematic review methodology. The working group met once a month from the inception of the review in July 2022 until its completion in May 2023. In the current paper, we have presented the findings regarding autistic adults and regarding mixed samples of adults and children and young people (CYP) populations in which only combined outcomes were reported. The findings for CYP will be reported in a separate paper.

### Search strategy

Three electronic databases – MEDLINE (01/01/1994-DATE), PsycINFO (01/01/1994-DATE) and CINHAL (01/01/1994-DATE) – and two pre-print servers – medRxiv and PsyArXiv – were searched, using a combination of keyword and subject heading searches of terms for autism spectrum disorders, services/treatments and mental health problems. The search was restricted to studies published since 1994, to cover the Diagnostic and Statistical Manual of Mental Disorders fourth and fifth edition periods. The reference lists of identified relevant systematic reviews were searched, and experts including academics and lived experience networks were contacted to identify relevant papers. The full search strategy can be found in Supplementary Tables 2–4.

### Inclusion and exclusion criteria

#### Population

For studies evaluating the effectiveness and/or acceptability/feasibility of treatment, we included adults (18+ years) and mixed samples of adults and CYP with an autism diagnosis, or who suspected they were autistic or who were identified by clinicians as potentially autistic. Perspectives of carers and clinicians providing treatment to this population were also included. In all studies apart from those that explored detection of autism in mental health services, we excluded those with samples that included both autistic people and non-autistic people, unless data were reported separately for the autistic group.

#### Strategies

We included any bespoke or adapted mental health intervention (pharmacological, non-pharmacological or combinations) specifically for autistic people receiving mental healthcare from specialist mental health services and/or in primary care. We included studies describing and characterising bespoke or adapted mental health interventions for autistic people, or reporting on adaptations intended to improve access, experiences of care and mental health outcomes or strategies to identify autism in mental health services. Bespoke interventions were defined as those that were reported as developed specifically for autistic people, whereas adapted mental health interventions were defined as existing interventions that were reported as adapted to meet the needs of autistic people. Studies with any kind of comparison group (e.g. standard care, bespoke interventions or adapted approaches) or without a comparison group were included.

#### Outcomes

We included any quantitative measure or qualitative account of feasibility (e.g. recruitment adherence and retention rates), service use (e.g. engagement), acceptability of care, and experiences and satisfaction with care at end of treatment or follow-up for the second review question (RQ2). We also included any quantitative measure of mental health, detection of autism, quality of life, service use (e.g. in-patient admission, acute crisis care) and social outcomes (e.g. social functioning) at end of treatment or follow-up for the third review question (RQ3). Studies measuring physical health outcomes only were excluded.

#### Study types

All study designs and service descriptions reporting acceptability and feasibility outcomes were eligible for RQ2. Only empirical quantitative study designs, including service evaluations and clinical audits, were eligible for RQ3. We excluded systematic or narrative reviews, small-N case studies, commentaries, book chapters, editorials, letters, conference abstracts and theses.

### Study selection

Two members of the review team (T.S., P.B.) piloted the selection strategy. Title and abstract screening was conducted by members of the review team (A.K., T.S., K.R.K.S., A.G., T.P., U.F.) with a random 10% of the search records independently reviewed in duplicate (T.P., S.L.) (inter-rater agreement 97.98%). The full text of eligible articles was then screened by members of the review team (T.P., A.G., A.K., T.S., D.S., K.R.K.S., S.L., R.C., J.G., H.B., U.F.) and independently reviewed in duplicate (T.P., S.L.), in accordance with Cochrane guidance.^22^ Conflicts were resolved by discussion and consultation with a third reviewer (S.J. or V.T.) and with the working group. In instances where the setting or the intervention were unclear, study authors were contacted to determine eligibility. Study selection was carried out in Rayyan (a web-based software).^23^

### Data extraction

Following study selection, members of the review team including lived experience researchers (T.P., A.G., A.K., T.S., D.S., U.F., S.L., J.G., H.B., A.T., M.M., G.B., R.C.) extracted the following: study design, aims, setting, sample size, participant characteristics (e.g. age, ethnicity, gender, diagnosis), outcome measures, strategies or adaptations (e.g. type and brief description) and relevant findings (feasibility, acceptability and effectiveness). The data extraction form was first piloted on 10% of the included studies (S.L., R.C.) and revised accordingly based on feedback from the working group. Data were extracted independently in duplicate, and consensus of the extracted data was achieved. Data extraction began on 16 September 2022.

In response to the observation by lived experience researchers working on the study that a bias was apparent in multiple studies, whereby researchers may have unknowingly made decisions, discussed concepts or analysed findings in a way that missed out or did not appropriately consider key elements of the autistic experience, a decision was made in the working group to pilot a method of assessing this. A lived experience researcher (R.R.O.) developed an Autism-Inclusive Research Assessment based on a combination of existing literature and personal experience to capture these important aspects of the included studies, which was piloted by members of the team (S.L., T.P.). The five criteria that made up this assessment were: (1) reported involvement from people with lived experience in the design, conduct or write-up of the study; (2) for studies with qualitative elements, reported adjustments made to the data collection process;^24^ (3) for studies with quantitative elements, reported adjustments made to the data collection tools;^25^ (4) for studies with quantitative elements, reported adaptations or validity of relevant outcome measures for autistic people; (5) for studies with quantitative elements, perceived focus of the tested intervention/strategy on masking/changing autistic traits which might have not inherently impacted quality of life or caused distress (e.g. use of outcome measures relating to social skills or explicitly seeking to reduce autism symptoms).^26^ Data for all criteria were extracted from the included studies by two researchers (A.G., A.K.), with lived experience researchers (R.J., J.P.) as second assessors of the final criterion.

### Quality assessment and certainty of evidence

The Mixed Methods Appraisal Tool (MMAT)^27^ was used to assess study quality. This is an established tool for evaluating quantitative, qualitative and mixed methods studies. Scores range from zero (low quality) to five (high quality). All scores were independently assessed in duplicate before reaching consensus. The Grading of Recommendations Assessment, Development and Evaluation (GRADE) system,^28^ adapted for narrative synthesis,^29^ was used to assess the strength of the evidence contributing to effectiveness outcomes. Two reviewers (T.P., S.L.) independently assessed the certainty of evidence and addressed inconsistencies before reaching consensus.

### Data synthesis

A narrative synthesis was undertaken following Economic and Social Research Council guidelines.^30^ The heterogeneity of study designs and strategies including various combinations of adaptations in the included studies informed the narrative approach to data synthesis and limited the extent to which data could be synthesised. All identified intervention-level and service-level adaptations were grouped based on shared commonalities (e.g. environmental adjustments, adapted communication) to develop top-level and sub-level categories. Category development was informed by the input of lived experience researchers. Two meetings were held with lived experience researchers, in addition to the monthly working groups meetings, to share their views on the generated categories. Lived experience researchers were also given the opportunity to provide written feedback. To present extracted data, articles were categorised by the type of strategy (i.e. adaptations to mental health interventions, service improvements/adaptations or bespoke strategies) and study design (i.e. randomised controlled trials (RCTs), non-randomised controlled trials, surveys, before-and-after comparisons and service evaluations). Extracted data related to the five criteria used in the Autism-Inclusive Research Assessment were synthesised descriptively. The review findings presented below have been refined and interpreted through discussions with and feedback from the working group.

## Results

**Fig. 1:**
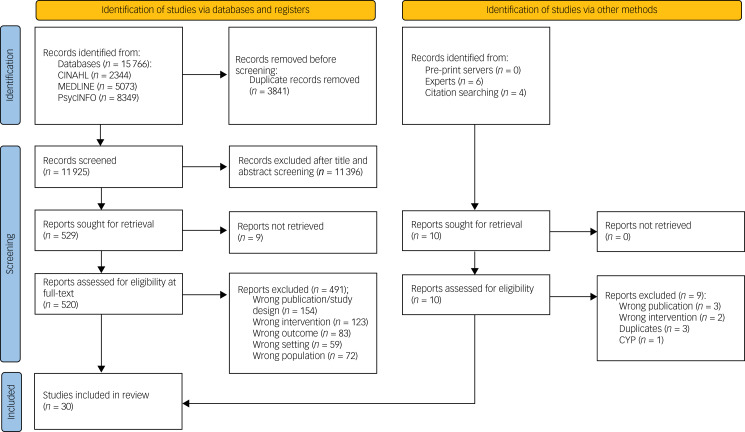
Preferred Reporting Items for Systematic Reviews and Metaanalyses flowchart. CYP, children and young people.

The PRISMA flow diagram is shown in Fig. 1. In total, 30 articles met the inclusion criteria. A list of included studies can be found in Supplementary Table 5.

### Study design

Of the 30 articles, two were RCTs,^31,32^ three were pilot RCTs,^33–35^ three were non-randomised controlled trials,^36–38^ three were qualitative,^39,40^ one was retrospective analytical cross-sectional,^41^ four were surveys,^42–45^ four were service evaluations^46–49^ and ten were before–after comparison studies.^50–59^ Two of these articles were from the same trial.^34,39^ Study characteristics are described in detail in Table 1 and Supplementary Table 6.

**Table 1:** Study characteristics (*N* = 30)

Author (Ref.)	Country	Study design	Population	Age mean (s.d.), range	Intervention	Setting	Condition targeted
Bemmer et al (2021)^50^	Australia	Pre-post	Autistic CYP and adults	22.77 (5.31), 16–38	Adapted group CBT for social anxiety	Research clinic within primary healthcare network and Headspace clinical services	Social anxiety
Blainey et al (2017)^46^	England	Service evaluation	Autistic adults	30 (10.64)^a^	Adapted individual CBT	Autism psychological therapies services	Psychological distress
Brugha et al (2020)^42^	England	Cross-sectional two-phase survey	Autistic adults	42.3% <40; 29.8% 40–49; 27.9% 50+years^a^^,^^b^	Autism screening tools: Autism-spectrum quotient and RAADS-R	In-patient, out-patient and community mental health services	Autism identification
Cooper at al. (2018)^43^	UK	Cross-sectional survey	Mental health staff working with autistic people	Not reported	Adapted CBT	IAPT and secondary mental health services	Mental health difficulties/Psychological distress
Dreiling et al (2022)^47^	USA	Service evaluation	Staff working with autistic people	42.22 (10.6), 25–66	Service strategy Project ECHO	Community services	Mental healthcare
Ekman et al (2015)^51^	Sweden	Quasi-experimental open pilot	Autistic CYP and adults	Teens: 14.9 (1.5), 13–17. Adults: 29.8 (4.4), 23–36	Adapted individual CBT for anxiety	Psychiatric clinic, treatment centre for youth and private clinic	Anxiety
Fisher at al. (2023)^44^	Netherlands; UK; Australia; USA; Egypt; Greece; New Zealand	Delphi Survey (three rounds)	Staff working with autistic people	Not reported	Adapted EMDR	Psychological therapies, community mental health, intellectual disability, forensic and tertiary services, independent practice, education, military, voluntary organisations	PTSD
Flygare et al (2020)^52^	Sweden	Non-randomised clinical effectiveness	Autistic adults	23.84 (5.90)	Adapted individual CBT for OCD	Specialist out-patient OCD clinic	OCD
Hare et al (2016)^53^	UK	Case series	Autistic adults	Not reported	Bespoke RTSM using a mobile platform	Unclear	Stress
Harrison at al (2020)^41^	USA	Retrospective cross-sectional	Autistic adults	33.33 (12.04), 18–66	Autism screening tool: ASD-DF derived from PAI	Autism out-patient clinic and in-patient psychiatric hospital	Autism identification
Helverschou et al (2021)^48^	Norway	Service evaluation	Autistic CYP and adults, intellectual disability	28.6 (10.6), 16–66	Service strategy: AUP network	Specialist hospital-level mental health services	Psychiatric problems
Horwood et al (2021)^39^	UK	Qualitative (part of a pilot RCT)	Autistic adults	40,^c^ 21–58	Adapted guided self-help CBT for depression	Autism services	Depression
Jones et al (2021)^45^	UK	Cross-sectional survey – service evaluation	Staff working with autistic people	Not reported	Service adaptations	In-patient units	Mental healthcare
Kiep et al (2015)^54^	Netherlands	Non-randomised	Autistic adults	37.9 (14)	Adapted group MBT-AS	Autism centre	Mental health difficulties
Langdon et al (2016)^33^	UK	Pilot single-blind RCT	Autistic CYP and adults	Combined: 35.9 (14.6), 17–65. Treatment: 33.1 (14.6), 20–64. Control: 38.7 (14.3), 17–65	Bespoke group CBT for anxiety	Autism, intellectual disability and adult mental health services	Anxiety
Lobregt-van Buuren et al (2019)^37^	Netherlands	Non-randomised add-on design	Autistic adults	34.48 (11.73)	Adapted individual EMDR	Out-patient/community services	PTSD
Maskey et al (2019)^55^	UK	Pilot study	Autistic adults	29.8,^c^ 18.8–57	Bespoke individual CBT for anxiety with virtual reality	Autism services, autism support network	Anxiety
McGillivray et al (2014)^36^	Australia	Quasi-experimental	Autistic CYP and adults	Treatment: 20.27 (4.39). Control: 20.50 (3.4) Both groups: 15–25	Bespoke group CBT for anxiety, stress and depression	Disability service agency	Mental health difficulties
Oshima et al (2021)^56^	Japan	Single-arm preliminary study	Autistic adults	26.8 (6.39), 20–39	Adapted individual schema therapy	University hospital	Mental health difficulties
Pahnke et al (2019)^57^	Sweden	Open pilot study	Autistic adults	49 (12), 25–65	Adapted group ACT	Psychiatric clinic	Mental health difficulties
Petty et al (2021)^40^	UK	Qualitative	Staff working with autistic people	25–44^b^	Service adaptations	Specialist autism service	Mental healthcare
Russell et al (2013)^31^	UK	Single-blind RCT	Autistic CYP and adults	Treatment: 28.6 (11.3), 14–49. Control: 25.2 (13.5), 14–65	Adapted individual CBT for OCD and adapted individual anxiety management	Specialist autism, OCD clinics and mental health services	OCD
Russell et al (2020)^34^	UK	Pilot single-blind RCT	Autistic adults	Treatment: 35.3 (13.6). Control: 42.2 (12.6)^a^	Adapted guided self-help CBT for depression	Autism services	Depression
Sizoo et al (2017)^38^	Netherlands	Non-randomised controlled	Autistic adults	Treatment: 35.1 (9.22). Control: 39.4 (10.81)^a^	Adapted group MBSR and adapted group CBT	Out-patient psychiatric clinic	Mental health difficulties
Spain et al (2017)^58^	UK	Non-randomised single arm	Autistic adults	31 (7.9), 22–48	Bespoke group CBT for group interaction anxiety and social skills	Autism out-patient psychological therapies service	Social anxiety
Spain et al (2017)^18^	UK	Qualitative	MDT professionals working with autistic people	Not reported	Adaptations to standard clinical approach	In-patient and out-patient services	Social anxiety
Spek et al (2013)^32^	Netherlands	RCT	Autistic adults	Control: 40.1 (11). Treatment: 44.4 (11.1)^a^	Adapted group MBT-AS	Autism centre	Mental health difficulties
Tchanturia et al (2021)^49^	UK	Service evaluation	Autistic adults	Not reported	Service strategy: PEACE pathway	In-patient wards for eating disorders	Mental healthcare
Wijker et al (2020)^35^	Netherlands	Exploratory single-blind RCT	Autistic adults	36% 18–32, 30% 33–46, 34% 47–60^b^	Bespoke individual AAT	Psychiatric out-patient service for autism	Stress
Wise et al (2019)^59^	USA	Open trial	Autistic CYP and adults	17.14 (1.68), 16–20	Adapted individual CBT for anxiety	University-based health clinic specialising in the treatment of anxiety	Anxiety

AAT, animal-assisted therapy; ACT, acceptance and commitment therapy; ASD-DF, autism spectrum disorder discriminant function; AUP, autism, intellectual disability and psychiatric disorder; CBT, cognitive–behavioural therapy; CYP, children and young people; ECHO, extension for community healthcare outcomes; EMDR, eye movement desensitisation and reprocessing; IAPT, improving access to psychological therapies; MBSR, mindfulness-based stress reduction; MBT-AS, mindfulness-based therapy for autism spectrum conditions; MDT, multidisciplinary team; OCD, obsessive-compulsive disorder; PAI, personality assessment inventory; PEACE, pathway for eating disorders and autism developed from clinical experience; PTSD, post-traumatic stress disorder; RAADS-R, Ritvo autism–Asperger's diagnostic scale-revised; RCT, randomised controlled trial; Ref., reference; RTSM, real-time stress management**.**

a. Range not reported.

b. Mean (s.d.) not reported.

c. No s.d. reported.

### Quality assessment

According to appraisal using the MMAT,^27^ 16 studies (four randomised controlled, six non-randomised, two quantitative descriptive, one mixed methods, three qualitative) were of high quality (≥4 criteria met), seven studies (four non-randomised, two mixed methods, one quantitative descriptive) were of moderate quality (three criteria met) and seven studies (six non-randomised, one mixed methods) were of low quality (≤2 criteria met). All MMAT ratings can be seen in Supplementary Table 7.

### Autism-Inclusive Research Assessment

Seven out of 30 studies (24%) reported that autistic people were involved in conducting the study. None of the eight studies with a qualitative element reported any adjustments to the data collection process (e.g. allowing non-verbal/non-oral communication). Two out of 27 studies (7%) with a quantitative element reported making some adjustments to the data collection tools (e.g. adapting Likert scales for greater precision, using straightforward language). Ten out of 27 studies (37%) with a quantitative element reported on the psychometric properties or adaptations of the relevant outcome measures to make them more appropriate for autistic individuals. Six out of the ten studies (60%) used at least one adapted or validated outcome measure relevant to the review, and the remaining four studies (40%) stated that the relevant outcome measures had not been validated or adapted specifically for autistic people. For five of the 21 studies (24%) with a quantitative element that measured outcomes in autistic mental health service users, the intervention/strategy was perceived to involve some focus on masking people's autistic traits. However, 13 of the 21 studies (62%) were not perceived to have any evidence to suggest such a focus, and this was unclear for three of the 21 studies (14%). See Supplementary Table 8 for all extracted data related to the Autism-Inclusive Research Assessment.

### Sample characteristics

Sample sizes at baseline were small across most studies, ranging from 7 to 1487 (median 103, *n* = 28 studies). Twenty-four studies included participants who were service users. All participants of these studies had a diagnosis of autism except for two studies relating to the detection of autism, which included people who had not been diagnosed as autistic at the time study data were obtained.^41,42^ Only one study included participants with intellectual disability.^48^

Twenty studies reported on co-occurring mental health conditions at baseline: obsessive-compulsive disorder (OCD),^31,52^ depression,^34,39^ anxiety,^33,55^ post-traumatic stress disorder (PTSD),^37^ stress,^35^ eating disorders^49^ and a combination of mental health difficulties.^32,36,38,41,42,46,51,54,56,57,59^ Seventeen papers reported on adults^32,34,35,37–39,41,42,46,49,52–58^ and seven papers reported on both CYP and adults.^31,33,36,48,50,51,59^ Six papers reported on staff perspectives of how to adapt and deliver better care for autistic people across the lifespan.^40,43–45,47^ Sample characteristics are described in detail in Supplementary Table 6.

### Types of strategies used to improve mental healthcare in autism

#### Intervention-level and service-level adaptations

Studies tended to use several adaptations; hence, most studies were found to be relevant to multiple categories. The following six top-level adaptation categories were identified: communication accommodations (*n* = 17), intervention content (*n* = 13), intervention structure (*n* = 9), increasing knowledge and detection of autism (*n* = 8), accommodating individual differences (*n* = 9) and environmental adjustments (*n* = 5). Table 2 and Supplementary Table 9 describe the adaptations in each study. Most studies reported a general rationale for adaptations (e.g. to address barriers and needs of autistic people). Individual adaptations often lacked a comprehensive description and rationale.

**Table 2:** All service-level and intervention-level adaptations (simplified version) (*N* = 24)

Top-level category	Sub-category	Summary	*N* studies
Increase knowledge and detection of autism	Clinician training and skills	Training to administer measures, tailor treatment to individual needs and increase self-efficacy, knowledge of autism and skills. Use of skills such as normalising experiences and prioritising therapeutic relationship.	5
	Introduce screening tools for the detection of autism	Use of assessments such as the Autism Spectrum Disorder-Discriminant Function (ASD-DF), the Autism-Spectrum Quotient and the Ritvo Autism–Asperger's Diagnostic Scale-Revised (RAADS-R).	3
Environmental adjustments	Provide environmental and practical adjustments	Provide adjustments to ensure consistency such as offering sessions at the same time and place. Minimise sensory distractions such as a low-stimulus area, adjustments to noise, decor, odour, lighting and meals.	5
	Normalise the use of sensory resources and stimming	Provide sensory materials such as ear defenders, weighted blankets, stress ball, relaxing music and sensory box. Encourage use of stimming behaviour.	4
Communication accommodations	Plan in advance	Share a plan in advance, ensure the client is prepared, find out about the client in advance, use appointment reminders, pay extra attention to planning and discuss issues.	5
	Clear communication	Provide clear instructions and guidance, repetition, be more directive, monitor, adapt and slow the pace of communication.	6
	Use simple and preferred language	Avoid using metaphors, abstract language, awareness of the language, use plain and preferred language.	11
	Use simple written material and visual aids	Use written information and external cues such as whiteboard, colour-coded worksheets, timers, agendas and calendars. Use visual aids such as drawings, pictures, videos and leaflets.	11
	Provide communication support	Host well-being groups and use communication passports and social stories.	2
Accommodate individual differences	Evaluate individual needs and preferences	Evaluate preferences, sensitivities, needs, likes and dislikes, coping strategies and daily habits.	5
	Encourage individual's hobbies and interests	Include and ask about the individual's special interests and hobbies in therapy.	4
	Tailor practice to individual needs and preferences	Tailor care plans and practice to individual differences such as incorporating approaches targeted at neurodevelopmental comorbidities, being flexible with the treatment manual and the session timings, and ensuring that resources are appropriate for the person's gender.	7
Intervention structure adaptations	Format of intervention	Reduce or increase the number and duration of sessions and exercises, additional support by therapists.	6
	Family/caregiver/other involvement	Involve important people such as family members, partners, teachers throughout therapy.	6
Intervention content adaptations	Simplify and structure content	Remove or simplify psychoeducation and cognitive elements and ensure that there is structure.	6
	Take it slow	Take a slow/progressive approach to treatment, regular breaks.	2
	Consider the role of autism	Consider the role of autism, develop an understanding of autism such as its characteristics and impact on daily life.	2
	Integrate emotion-focused strategies	Provide psychoeducation on emotions, arousal and feeling physiologically overwhelmed, and exercises to access emotions.	6
	Integrate cognitive–behavioural approaches	Provide cognitive and behavioural strategies including building a positive self-image, coping strategies and making links between behaviour, thoughts and feelings.	6
	Integrate social skills training	Integrate social skills training such as entering and maintaining conversations and managing disagreements.	3

Sixteen articles described studies of mental health interventions that had been adapted to make them more appropriate for autistic people. These included adaptations of cognitive–behavioural therapy (CBT) for anxiety,^50,51,59^ CBT for OCD,^31,52^ mindfulness-based therapy for autism spectrum disorders (MBT-AS),^32,54^ eye movement desensitisation and reprocessing (EMDR),^37,44^ guided self-help CBT for depression,^34,39^ acceptance and commitment therapy (ACT),^57^ Schema therapy,^56^ mindfulness-based stress reduction (MBSR),^38^ CBT for anxiety and depression^38^ and CBT aimed to reduce general psychological distress.^43,46^ No trials directly compared adapted and non-adapted mental health interventions; hence, no conclusions could be drawn as to whether specific adaptations resulted in better outcomes.

Eight articles described studies investigating service adaptations, largely related to autism-specific training of staff and environmental adjustments. These studies examined clinical pathways,^49^ models,^47^ networks^48^ and general adaptations^18,40,45^ to improve quality of mental healthcare for autistic people, and initiatives to improve the detection of autism.^41,42^

#### Bespoke mental health interventions

Six studies examined bespoke mental health interventions designed for autistic people. These were individual real-time stress management (RTSM) using a mobile platform,^53^ CBT for anxiety,^33^ CBT for social anxiety,^58^ CBT for anxiety in combination with virtual reality,^55^ CBT for anxiety, stress and depression^36^ and animal-assisted therapy (AAT).^35^

### Evaluation of strategies used to improve mental healthcare in autism

#### Certainty of evidence for effectiveness of strategies

Results from 23 studies contributed to the GRADE assessment. The certainty of evidence for the effectiveness of strategies (*n* = 19), as rated using the GRADE system,^28^ ranged from very low to moderate (Supplementary Table 10). No strategies were assessed to have high-certainty evidence for effectiveness, and only three out of 19 (16%) strategies were deemed to have moderate-certainty effectiveness evidence, i.e. adapted individual CBT interventions for anxiety^51,59^ and for OCD,^31,52^ and strategies for detection of autism.^41,42^ Certainty of evidence for effectiveness was generally very low or low for the remaining included strategies (53% and 32%, respectively).

#### Adaptations to mental health interventions

Overall, most adapted mental health interventions were evaluated as feasible and acceptable, except for one study of CBT for OCD, which showed limited feasibility.^52^ Individual adaptations were largely viewed positively,^39,51^ and clinicians reported frequently used adaptations to CBT^43^ and EMDR.^44^ Evidence on effectiveness was inconclusive as RCTs were not sufficiently powered, and most were before-and-after comparison studies. The main findings of adapted mental health interventions can be found in Tables 3 and 4, and detailed results of individual studies are available in Supplementary Table 11.

**Table 3:** Main findings of adaptations to individual mental health interventions

Study design (*N*) (Ref.)	Study characteristics (*N*)	Participant characteristics (*N*)	Strategy (*v.* comparison) (*N*)	Adaptation categories and sub-categories (*N*)	Main findings and certainty of evidence
Adapted individual CBT for anxiety					
Before-and-after comparison (2)^51,59^	Sample size: 7–18 (2). Country: USA (1); Sweden (1). Setting: Psychiatric clinic, treatment centre for youth and private clinic (1); university-based health clinic specialising in the treatment of anxiety (1).	Diagnoses: ASC (2); anxiety (2). Demographics: Adults (1); CYP and adults (1); 39–57% male (2); 14% ‘more than one race’ (1). Participants: Service users (2).	Individual adapted CBT for anxiety (2).	**Communication accommodations (2):** Use simple, written material and visual aids (2). **Accommodate individual differences (1):** Encourage individual's hobbies and interests (1). **Intervention structure (1):** Family/caregiver/other involvement (1). **Intervention content (1):** Integrate emotion-focused strategies (1); integrate cognitive–behavioural approaches (1); integrate social skills training (1).	**Acceptability:** Use of visualisation was perceived as helpful by participants (1). **Feasibility:** Low drop-out rate (1). **Effectiveness:** Significant pre-post improvements in general psychopathology (1), self-reported anxiety (1) and clinician-rated anxiety (1) as well as in global functioning (1), but not in depression and self-reported anxiety (1). **Certainty of evidence (2):** Low.
Adapted individual CBT for OCD					
RCT (1); Before-and-after comparison (1)^31,52^	Sample size: 19–46 (2). Country: UK (1); Sweden (1). Setting: Specialist out-patient OCD clinic (1); specialist autism, OCD clinics and mental health services (1).	Diagnoses: ASC (2); OCD (2). Demographics: Adults (1); CYP and adults (1); 42–70% male (2). Participants: Service users (2).	Individual CBT for OCD *v.* anxiety management (1); Individual CBT for OCD (1).	**Communication accommodations (2):** Clear communication (1); use simple and preferred language (2); use simple, written material and visual aids (2). **Accommodate individual differences (1):** Evaluate individual needs and preferences (1); encourage individual's hobbies and interests (1). **Intervention structure (1):** Family/caregiver/other involvement (1). **Intervention content (2):** Integrate emotion-focused strategies (2); simplify and structure content (1); integrate cognitive–behavioural approaches (1).	**Acceptability:** No significant differences in treatment satisfaction between adapted CBT for OCD and anxiety management. Significantly more participants in the AM group requested crossover to the CBT group than vice versa (1), good homework compliance (1). **Feasibility:** High attendance rate (1), low drop-out rate (2), low attendance rate (1) **Effectiveness:** Adapted CBT for OCD was not superior to AM group on primary (clinician-rated OCD) and secondary (psychopathology, self/parent-reported OCD, depression, anxiety, social anxiety, and social functioning) outcomes (1). Significant pre-post improvements in OCD and depression (1). No significant change in social outcomes and quality of life over time (1). **Certainty of evidence (2):** Moderate.
Adapted EMDR					
Non-randomised controlled trial (1); Survey (1)^37,44^	Sample size: 27 (1), 103 (1). Country: Netherlands; UK; Australia; USA; Egypt; Greece; New Zealand (1); Netherlands (1). Setting: Mixed mental healthcare setting (1); out-patient/community services (1).	Diagnoses: ASC (1); PTSD (1). Demographics: Adults (1); 62% male (1). Participants: Staff working with autistic individuals across the lifespan (1), service users (1).	EMDR + TAU *v.* TAU (1); EMDR (1).	**Communication accommodations (2):** Use simple and preferred language (2); plan in advance (1); clear communication (1); use simple, written material and visual aids (1). **Increase knowledge and detection of autism (1):** Clinician training and skills (1). **Environmental adjustments (1):** Provide environmental and practical adjustments (1); normalise the use of sensory resources and stimming (1). **Accommodate individual differences (1):** Evaluate individual needs and preferences (1); encourage individual's hobbies and interests (1); tailor practice to individual needs and preferences (1). **Intervention structure (1):** Family/caregiver/other involvement (1). **Intervention content (1):** Take it slow (1); consider the role of autism (1); integrate emotion-focused strategies (1); integrate cognitive–behavioural approaches (1).	**Acceptability:** EMDR sessions were perceived as stressful, but a high majority would choose to do it again (1). **Feasibility:** Most adaptations were frequently used by staff across studies (1); low drop-out rate (1). **Effectiveness:** Significant pre-post improvements in PTSD and psychological distress (1). **Certainty of evidence (1):** Low.
Adapted Schema therapy					
Before-and-after comparison (1)^56^	Sample size: 12 (1). Country: Japan (1). Setting: University hospital (1).	Diagnoses: ASC (1). Demographics: Adults (1); 50% male (1). Participants: Service users (1).	Schema therapy (1).	**Intervention content (1):** Consider the role of autism (1).	**Feasibility:** Low drop-out rate (1). **Effectiveness:** Significant pre-post improvements in global functioning, but not in quality of life as primary outcomes, and no significant pre-post improvements in secondary outcomes (depression, anxiety, social anxiety and OCD) (1). **Certainty of evidence (1):** Very low.
Adapted individual CBT for general psychological distress					
Before-and-after comparison (1); Survey (1)^43,46^	Sample size: 50–81 (2). Country: UK (2). Setting: Autism psychological therapies services (1); IAPT and secondary mental health services (1).	Diagnoses: ASC (1). Demographics: Adults (1); 74.1% male (1); 40.7% Black and minority ethnic British (1). Participants: Staff working with autistic individuals across the lifespan (1), Service users (1).	CBT (2).	**Communication accommodations (2):** Use simple, written material and visual aids (2); clear communication (1); use simple and preferred language (1). **Accommodate individual differences (2):** Tailor practice to individual needs and preferences (1); encourage individual's hobbies and interests (1). **Intervention structure (2):** Format of intervention (2); Family/caregiver/other involvement (2). **Intervention content (2):** Integrate social skills training (1); simplify and structure content (1); integrate emotion-focused strategies (1); integrate cognitive–behavioural approaches (1).	**Feasibility:** High attendance rate (1); most reported adaptations were frequently used by staff, and adaptations that were used less consistently included: shorter or longer sessions, avoidance of metaphors in therapy, involving a family member or partner in sessions, use of cognitive strategies (1). **Effectiveness:** Significant pre-post improvements in global distress (1). **Certainty of evidence (1):** Very low.
Adapted guided self-help CBT for depression					
RCT (1); Qualitative (1)^34,39^^a^	Sample size: 26–70 (2). Country: UK (2). Setting: Autism services (1).	Diagnoses: ASC (2); depression (2). Demographics: Adults (2), 69–81% male (2); 0–6% Black and minority ethnic (2) Participants: Service users only (1); service users and staff (1).	Guided self-help for depression *v.* TAU (2).	**Communication accommodations (1):** Plan in advance; use simple, written material and visual aids (1). **Accommodate individual differences (1):** Evaluate individual needs and preferences (1); tailor practice to individual needs and preferences (1). **Intervention structure (1):** Format of intervention (1) **Intervention content (1):** Simplify and structure content (1); integrate cognitive-behavioural approaches (1).	**Acceptability:** Guided self-help CBT was viewed positively by participants (1). **Feasibility:** High attendance rate (1); low drop-out rate (1).

ASC, autism spectrum condition; CBT, cognitive–behavioural therapy; EMDR, eye movement desensitisation and reprocessing; IAPT, improving access to psychological therapies; OCD, obsessive-compulsive disorder; PTSD, post-traumatic stress disorder; Ref., reference.

a. Two articles were from the same pilot randomised controlled trial, one of which was a qualitative study examining the acceptability of guided self-help CBT for depression.

**Table 4:** Main findings of adaptations to group mental health interventions

Study design (*N*) (Ref.)	Study characteristics (*N*)	Participant characteristics (*N*)	Strategy (*v.* comparison) (*N*)	Adaptation categories and sub-categories (*N*)	Main findings and certainty of evidence
Adapted group MBT-AS					
RCT (1); non-randomised controlled trial (1); before-and-after comparison (1)^32,38,54^	Sample size: 42–59 (3). Country: Netherlands (3). Setting: Autism services (2); out-patient psychiatric clinic (1).	Diagnoses: ASC (3); depression and/or anxiety (3). Demographics: Adults (3), 59–70% male (3). Participants: Service users (3).	Group MBT- Autism-spectrum quotient *v.* waiting list (1); group MBT- Autism-spectrum quotient (1); group CBT for anxiety and depression *v.* Group MBSR (1).	**Communication accommodations (3):** Plan in advance (2); use simple and preferred language (3); clear communication (1). **Intervention structure (2):** Format of intervention (2). **Intervention content (3):** Simplify and structure content (2); take it slow (1).	**Feasibility:** High attendance rate (2); low drop-out rate (2). **Effectiveness:** Compared to waiting list, participants receiving MBT significantly improved in anxiety, depression, rumination and positive affect (1). Participants in both adapted CBT and adapted MBSR significantly improved in depression, anxiety, positive and negative affect and rumination, and there was no significant difference between treatment groups (1). Significant pre-post improvements in psychological problems, rumination and positive affect which remained stable over a 9-week period post-treatment (1). **Certainty of evidence (3):** Moderate.
Adapted group CBT for anxiety and depression					
Non-randomised controlled trial (1)^38^	Sample size: 59 (1). Country: Netherlands (1). Setting: Out-patient psychiatric clinic (1).	Diagnoses: ASC (1); depression and/or anxiety (1). Demographics: Adults (1), 59–70% male (1). Participants: Service users (1).	Group CBT for anxiety and depression *v.* Group MBSR (1).	**Communication accommodations (1):** Plan in advance; clear communication (1); use simple and preferred language (1). **Intervention content (1):** Take it slow (1).	**Effectiveness:** Participants in both adapted CBT and adapted MBSR significantly improved in in depression, anxiety, positive and negative affect and rumination, and there was no significant difference between treatment groups (1). **Certainty of evidence (1):** Very low.
Adapted group CBT for social anxiety					
Before-and-after comparison (1)^50^	Sample size: 84 (1). Country: Australia (1). Setting: Research clinic within primary healthcare network and Headspace clinical services (1).	Diagnoses: ASC (1). Demographics: Autistic CYP and adults(1); 60% male (1). Participants: Service users (1).	Group CBT for social anxiety (1).	**Intervention content (1):** Simplify and structure content; integrate cognitive–behavioural approaches (1); integrate social skills training (1).	**Acceptability:** High enjoyment, positive engagement, though some reported difficulties with a group member who was perceived as disruptive (1). **Feasibility:** Low drop-out rate and high attendance rate (1). **Effectiveness:** Significant pre-post improvements in anxiety and avoidance of social situations (primary outcomes) and social anxiety related to having conversations, depression, anxiety and stress (secondary outcomes), but not in social anxiety related to fears of being evaluated in daily activities and psychological distress (secondary outcomes) (1). **Certainty of evidence (1):** Very low.
Adapted group ACT					
Before-and-after comparison (1)^57^	Sample size: 10 (1). Country: Sweden (1). Setting: Psychiatric clinic (1).	Diagnoses: ASC (1). Demographics: Autistic adults (1); 50% male (1). Participants: Service users (1).	Group ACT (1).	**Communication accommodations (1):** Clear communication (1); use simple and preferred language (1); use simple, written material and visual aids (1). **Intervention structure (1):** Format of intervention (1). **Intervention content (1):** Integrate emotion-focused strategies (1).	**Acceptability:** High treatment credibility (1). Feasibility: High attendance rate, low drop-out rate, good homework compliance (1). **Effectiveness:** Significant pre-post improvements in stress, depression and in disability in social life, but not in anxiety, quality of life, disability at work/school and family life (1). **Certainty of evidence (1):** Very low.

ACT, acceptance and commitment therapy; ASC, autism spectrum condition; CBT, cognitive–behavioural therapy; MBSR, mindfulness-based stress reduction; MBT-AS, mindfulness-based therapy for autism spectrum disorders; Ref., reference.

#### RCTs and pilot RCTs

Three trials, including one pilot, evaluated adapted mental health interventions. One RCT found that compared to the waiting list, adapted group MBT-AS led to statistically significant improvements in all mental health outcomes including depression, anxiety, positive affect and rumination.^32^ The intervention also had a low drop-out rate and a high attendance rate. Another RCT found no statistically significant differences in treatment satisfaction and in primary (clinician-assessed OCD symptoms) and secondary (self-reported OCD symptoms, anxiety and depression) mental health outcomes between adapted individual CBT for OCD and adapted individual anxiety management post-treatment, apart from parent-report OCD symptoms that statistically significantly reduced only in the anxiety management group over time.^31^ Attendance rates were higher in the CBT arm; however, drop-out rates were similar in both groups. Neither RCT was sufficiently powered to demonstrate an effect. A pilot RCT comparing adapted individual guided self-help CBT for depression and treatment as usual (TAU) found that the former had a lower drop-out rate, while also achieving an acceptable attendance rate.^34^ A subsequent qualitative study^39^ using a subset of the sample demonstrated that the intervention was viewed positively by most participants. However, there were differing views about the pacing of the sessions and the use of predefined visual tools.

#### Non-randomised controlled trials

Two non-randomised controlled trials evaluated adapted mental health interventions. A study comparing the effectiveness of adapted group MBSR and adapted group CBT for anxiety and depression reported no statistically significant differences in mental health outcomes of anxiety, depression, positive and negative general mood and rumination at post-treatment.^38^ Both MBSR and CBT were adapted in the same way (see Table 4 and Supplementary Table 9). A study with a non-randomised add-on design reported statistically significant improvements in PTSD symptoms and psychological distress following EMDR + TAU compared to TAU only.^37^ The effect remained stable at 6–8 weeks follow-up. The study showed a low drop-out rate and, although all participants found EMDR sessions stressful, most indicated that they would choose the therapy again.

#### Surveys investigating perspectives of staff

Two surveys examined the perspectives of staff of adapted interventions to improve mental healthcare for autistic people. One survey found that most adaptations to CBT targeting psychological distress (e.g. use of plain English, structured and concrete approach) were highly endorsed by therapists, while others appeared to be used less consistently (e.g. shorter or longer sessions, avoidance of metaphors).^43^ Findings from a Delphi survey reported an array of adaptations identified by therapists as always, often or sometimes incorporated in EMDR.^44^ These included environmental adjustments, normalising experiences, communicating clearly, being flexible with the treatment manual, taking a slow approach and considering the role of autism within conceptualisation.

#### Before-and-after comparison studies

Eight before-and-after comparison studies examined adapted mental health interventions. Statistically significant improvements in outcomes over time were reported in all studies (Tables 3 and 4). However, causality cannot be inferred as there were no comparison groups, thus these will not be reported in detail.

A pre-post study of group CBT for social anxiety reported a low drop-out rate, high attendance rate and high participant enjoyment.^50^ Another study found that group MBT-AS had acceptable attendance and drop-out rates.^54^ Additionally, high levels of attendance, retention, homework compliance and treatment credibility were reported regarding group ACT^57^ One study reported a high attendance rate to adapted CBT for anxiety.^46^ A study of CBT for anxiety^59^ and a study of schema therapy^56^ reported low drop-out rates. Most participants found the adaptations (i.e. use of visualisation) of CBT for anxiety to be helpful.^51^ The drop-out rate of CBT for OCD was low, and homework compliance was adequate to good; however, the attendance rate was low.^52^

#### Mental health service adaptations

Overall, strategies to improve clinicians’ knowledge of autism and provide environmental adjustments in services were evaluated as acceptable and feasible.^47–49^ Several service adaptations were reported as frequently implemented in services by staff.^40,45^ Additionally, self-report tools were found to discriminate between autistic and non-autistic people.^41,42^ Table 5 presents findings of evaluations of strategies at service level intended to improve care.

**Table 5:** Main findings of mental health service adaptations

Study design (*N*) (Ref.)	Study characteristics (*N*)	Participant characteristics (*N*)	Intervention/Strategy (*v.* comparison) (*N*)	Adaptation categories and sub-categories (*N*)	Main findings and certainty of evidence
Detection of autism in mental healthcare					
Survey (1); Retrospective analytical cross-sectional (1)^41,42^	Sample size: 738 (1); 1487 (1). Date: 2020 (2). Country: England (1); USA (1). Setting: Secondary mental health services (in-patient, out-patient and community) (1); autism out-patient and mental health in-patient services (1).	Diagnoses: 1–11.4% clinical ASC diagnosis (2); mixed mental health clinical diagnoses (2). Demographics: Adults (2); 49.6–64% male (2); 25% Black and minority ethnic (1). Participants: Service users (2).	Service-level strategy (detection of autism): autism-spectrum quotient and the RAADS-R (1); ASD-DF derived from the PAI in autism sample *v.* contrast in-patient sample and PAI clinical standardised sample (1).	**Increase knowledge and detection of autism (2):** Introduce screening tools for the detection of autism (2).	**Feasibility:** 66% response rate for either or both autism-spectrum quotient/RAADS-R, with the Autism-spectrum quotient appearing to be easier to complete compared to RAADS-R; however, some mental health services users with capacity showed difficulty with the self-completion questionnaires (1). **Effectiveness:** Self-report tools - the Autism-spectrum quotient, the RAADS-R (1) and the ASD-DF (1) - can identify fairly accurately individuals for whom further specialised autism assessment is warranted. **Certainty of evidence (2):** Moderate.
PEACE pathway					
Non-randomised controlled trial (1)^49^	Sample size: not reported (1). Country: UK (1). Setting: In-patient wards for eating disorders (1).	Diagnosis: ASC (1); eating disorders (1). Demographics: not reported (1). Participants: service users (1).	Service improvement: Pathway to improve care for autistic people with co-occurring eating disorders and non-autistic people with eating disorders (1).	**Increase knowledge and detection of autism (1):** Clinician training and skills (1). **Environmental adjustments (1):** Provide environmental and practical adjustments (1); normalise the use of sensory resources and stimming (1). **Communication accommodations (1):** Provide communication support (1).	**Effectiveness:** Reduction in the average cost and duration of admissions for autistic individuals with co-occurring eating disorders than non-autistic individuals with eating disorders. Increase in confidence of clinicians in supporting autistic individuals with eating disorders (1). **Certainty of evidence (1):** Low.
AUP network					
Before-and-after comparison (1)^48^	Sample size: 132 (1). Country: Norway (1). Setting: Specialist hospital-level mental health services (1).	Diagnosis: ASC (1); intellectual disability; co-occurring mental health difficulties (1). Demographics: CYP and adults (1); 67% male (1). Participants: Service users (1).	Service improvement: Network to improve access to and quality of tailored services for autistic adults with ID (1).	**Increase knowledge and detection of autism (1):** Clinician training and skills (1).	**Effectiveness:** Significant improvements in proportion with psychiatric disorders from referral to after 12 months, but not from after 12 months to 24–27 months (1). **Certainty of evidence (1):** Very low.
Project ECHO					
Before-and-after comparison (1)^47^	Sample size: 86 (1). Country: USA (1). Setting: Community services (1).	Demographics: 6% male (1); 14% Black and minority ethnic (1). Participants: mental health providers working with autistic people across the lifespan (1).	Service improvement: Project to increase knowledge of autism and co-occurring mental health difficulties in mental health providers (1).	**Increase knowledge and detection of autism (1):** Clinician training and skills (1).	**Acceptability:** High satisfaction (1). **Feasibility:** High attendance rate (1). **Effectiveness:** Significant pre-post improvements in self-efficacy, autism knowledge and awareness in best-practice treatment considerations for autistic people (1). **Certainty of evidence (1):** Very low.
General adaptations to standard practice					
Survey (1); qualitative (2)^18,40,45^	Sample size: 15–90 (3). Country: UK (3) Setting: In-patient units (1), specialist autism service (1), in-patient and out-patient services (1)	Participants: Staff working with autistic people across the lifespan (3)	Service adaptations: Evaluation of general strategies and adaptations to improve care (3)	**Increase knowledge and detection of autism (2):** Introduce screening tools for the detection of autism (1); clinician training and skills (2). **Environmental adjustments (3):** Provide environmental and practical adjustments (3); normalise the use of sensory resources and stimming (2). **Communication accommodations (3):** Plan in advance (1); clear communication (2); use simple and preferred language (2); use simple, written material and visual aids (2); provide communication support (1). **Accommodate individual differences (3):** Evaluate individual needs and preferences (2); tailor practice to individual needs and preferences (3).	**Feasibility:** Use of assessments and open access/on request low-stimulus area and the ability to adapt meal plans (in over 50% of units), lighting adaptations, scheduled access low-stimulus area and the use of specific protocols were used less frequently (in less than 50% of units) (1); Environmental adjustments, communication modifications, and consideration of gender were frequently used by staff and identified as important (1); Use of environmental and communication modifications, offer appointments at convenient times and encourage people to take an active role in their care (1).

ASC, autism spectrum condition; ASD-DF, autism spectrum disorder discriminant function; AUP, autism, intellectual disability and psychiatric disorder; ECHO, extension for community healthcare outcomes; PAI, personality assessment inventory; PEACE, pathway for eating disorders and autism developed from clinical experience; RAADS-R, Ritvo autism–Asperger's diagnostic scale-revised; Ref., reference.

#### Service evaluations

Three studies examined clinical pathways, models and networks to improve mental healthcare for autistic people. The Pathway for Eating Disorders and Autism developed from Clinical Experience (PEACE) aimed to introduce autism-specific training, create an autism-friendly ward and support sensory difficulties and communication.^49^ The pathway resulted in more reductions in the cost and average duration of hospital admissions in autistic individuals with eating disorders than non-autistic individuals with eating disorders. Evaluation of the PEACE pathway suggested that clinicians’ confidence in supporting autistic people with co-occurring eating disorders increased following its implementation.

Project Extension for Community Healthcare Outcomes (ECHO) utilised a tele-mentoring platform to connect primary care providers, to increase knowledge of autism and co-occurring mental health difficulties and to appropriately adapt treatments.^47^ Project ECHO was attended by most mental health providers, and increased knowledge, self-efficacy and awareness in best-practice treatment considerations for autistic individuals were reported post-ECHO sessions. The project was also viewed positively and was rated highly on satisfaction.

Another study reported statistically significant improvements in the proportion of psychiatric disorders from referral to after 12 months, which were sustained from 12 months to 24–27 months post-implementation of the Autism Intellectual Disability and Psychiatric Disorder (AUP) network.^48^ The AUP network aimed to improve access and quality of tailored services for autistic adults with intellectual disability and increase clinicians’ knowledge of how mental health difficulties present in autistic people.

#### Perspectives of staff

Three studies identified adaptations made to the service or their standard practice. One survey reported that the most frequent adaptations within in-patient units involved the use of assessments and open access/on request low-stimulus area, and the ability to adapt meal plans (over 50% of units), whereas lighting adaptation, scheduled access low-stimulus area and the use of specific protocols were used less frequently (less than 50% of units).^45^ Another qualitative study also reported a range of adaptations identified by staff, including environmental adjustments, communication modifications and consideration of gender, the majority of which were frequently used and perceived as important.^40^ A final qualitative study reported modifications that were made by multidisciplinary team professionals to their standard practice.^18^ These involved environmental and communication accommodations, offering appointments at convenient times and encouraging people to take an active role in their care.

#### Detection of autism

Two studies involved application in routine settings of autism screening instruments with the aim of improving detection within mental health services. One survey found that the autism-spectrum quotient and the Ritvo autism–Asperger's diagnostic scale-revised (RAADS-R) can identify fairly accurately autistic individuals from in-patient, out-patient and community mental health services.^42^ There was a good response rate for either or both autism-spectrum quotient and RAADS-R; however, it was highlighted that some mental health service users struggled with self-completion of the questionnaires, and the autism-spectrum quotient appeared to be easier to complete.^42^ A retrospective study reported that an Autism Spectrum Disorder Discriminant Function (ASD-DF) derived from the Personality Assessment Inventory can discriminate between a sample of autistic participants from a contrasting in-patient sample.^41^

#### Bespoke strategies to improve mental healthcare for autistic people

Most bespoke mental health interventions were evaluated as feasible and acceptable, apart from one study of RTSM,^52^ which demonstrated limited feasibility and acceptability. Evidence on effectiveness was inconclusive. Table 6 presents findings of evaluations of bespoke mental health interventions designed for autistic people.

**Table 6:** Main findings of bespoke strategies to improve mental healthcare for autistic people

Study design (*N*) (Ref.)	Study characteristics (*N*)	Participant characteristics (*N*)	Intervention (*v.* comparison) (*N*)	Main findings and certainty of evidence
Bespoke individual CBT for anxiety with virtual reality				
Before-and-after comparison (1)^55^	Sample size: 8 (1). Country: UK (1). Setting: Autism services, autism support network (1).	Diagnosis: ASC (1); anxiety and/or depression (1). Demographics: Adults (1); 50% male (1). Participants: service users (1).	Bespoke CBT for anxiety in combination with virtual reality (1).	**Feasibility:** High attendance rate, low drop-out rate (1). **Effectiveness:** 5 of the 8 participants were classified as treatment responders (1). **Certainty of evidence (1):** Very low.
Bespoke individual AAT				
RCT pilot (1)^35^	Sample size: 53 (1). Country: Netherlands (1). Setting: Psychiatric out-patient autism service (1).	Diagnosis: ASC diagnosis; stress. Demographics: Adults; 55% male. Participants: service users.	Bespoke AAT *v.* waiting list (1).	**Feasibility:** High attendance rate, low drop-out rate (1). **Effectiveness:** No significant differences between groups in perceived stress, general psychopathology symptoms and self-esteem (1). **Certainty of evidence (1):** Low.
Bespoke individual RTSM using a mobile platform				
Before-and-after comparison (1)^53^	Sample size: 14 (1). Country: UK (1). Setting: Unclear (1).	Diagnosis: High-functioning autism (1). Demographics: Adults (1); 55% men (1). Participants: Service users (1)	Bespoke RTSM using a mobile platform (1).	**Acceptability:** Limited acceptability (frustration and interference with daily life) (1). **Feasibility:** Low drop-out rate but engagement rate was also low (1). **Effectiveness:** Significant pre-post improvements in aggregated mean anxiety from baseline to post, but not in self-report anxiety and depression (1). **Certainty of evidence (1):** Very low.
Bespoke group CBT for social anxiety				
Before-and-after comparison (1)^58^	Sample size: 18 (1). Country: UK (1). Setting: Autism out-patient psychological therapies service (1).	Diagnosis: ASC (1). Demographics: Adults (1); 100% male (1); 17% Black and minority ethnic (1). Participants: Service users (1).	Bespoke CBT for social anxiety (1).	**Acceptability:** Participants found the intervention helpful; some would have preferred to be given additional practical strategies (1). **Feasibility:** High attendance rate, low drop-out rate (1). **Effectiveness:** Significant pre-post improvements in social anxiety, but not in anxiety, depression or social functioning (1). **Certainty of evidence (1):** Very low.
Bespoke group CBT for anxiety				
RCT pilot (1)^33^	Sample size: 52 (1). Country: UK (1). Setting: Autism, ID and adult mental health services (1).	Diagnosis: Asperger's syndrome, high-functioning autism or PDD-NOS (1); anxiety (1). Demographics: CYP and adults (1); 52% male (1); 98% White (1). Participants: Service users (1).	Bespoke CBT for anxiety + TAU *v.* waiting list (1).	**Acceptability:** Participants found the intervention helpful; some noted the need for longer sessions and the possibility of individual delivery (1). **Feasibility:** high attendance rate, low drop-out rate (1). **Effectiveness:** No significant differences between groups in primary (clinician-assessed anxiety) and secondary outcomes (self-report anxiety, depression, social phobia, functioning) (1). **Certainty of evidence (1):** Low.
Bespoke group CBT for anxiety, stress and depression				
Non-randomised controlled trial (1)^36^	Sample size: 42 (1). Country: Australia (1). Setting: Disability service agency (1).	Diagnosis: Asperger's syndrome or high-functioning autism (1); depressed mood, anxiety, stress and/or negative automatic thoughts (1). Demographics: CYP and adults (1); 73–81% male (1). Participants: Service users (1).	Bespoke CBT for anxiety, stress and depression vs waiting list (1).	**Feasibility:** Low drop-out rate, attendance rate is unclear (1). **Effectiveness:** No significant differences between groups in mental health outcomes, but those who scored higher in anxiety, depression and stress benefited the most (1). **Certainty of evidence (1):** Low.

AAT, animal-assisted therapy; ASC, autism spectrum condition; CBT, cognitive–behavioural therapy; CYP, children and young people; PDD-NOS, pervasive developmental disorder – not otherwise specified; Ref., reference; RTSM, real-time stress management; TAU, treatment as usual.

#### Pilot RCTs

Two pilot RCTs evaluated bespoke mental health interventions. One pilot crossover trial reported no statistically significant differences between the CBT group and the waiting list group for anxiety, in primary (clinician-assessed anxiety) and secondary mental health and social outcomes (self-report anxiety, depression, social phobia, functioning).^33^ Additionally, attendance and drop-out rates in the CBT group were acceptable, and reports from participants showed that the intervention was found to be helpful.^33^ Specifically, CBT participants felt supported by others, found listening to others’ problems helpful and experienced less anxiety. Participants also reported enjoying the interaction with others during the sessions. However, they noted the need for longer sessions, and it was suggested that individual rather than group-based delivery might be positive. Another pilot trial, comparing individual AAT with a waiting list, reported no statistically significant differences between the two groups in all mental health outcomes; however, the intervention had acceptable attendance and drop-out rates.^35^ Findings on effectiveness in both trials were inconclusive as these were not sufficiently powered to show an effect.

#### Non-randomised controlled trials

Only one non-randomised controlled trial evaluated bespoke mental health interventions. No statistically significant differences between the CBT group and the waiting list group were found, but subsequent analysis showed that participants who scored higher on measures of depression, anxiety and stress benefited the most from the intervention.^36^ The study also reported a low drop-out rate, but the attendance rate could not be determined as it was unclear how many completed the intervention.^36^

#### Before-and-after comparison studies

Three before-and-after comparison studies investigated bespoke mental health interventions. Statistically significant improvements in outcomes over time were reported (Table 6). However, causality cannot be inferred as there were no comparison groups; hence, these will not be reported in detail.

A study of individual CBT for anxiety in combination with virtual reality reported low drop-out rates.^55^ Similarly, a study of group CBT for social anxiety reported acceptable attendance and drop-out rates.^58^ Qualitative reports from participants indicated that the intervention helped them to meet others and feel more confident in social situations, while some stated that they would have benefited from additional practical strategies. Another study examining the use of RTSM reported an acceptable drop-out rate, but the engagement rate was low.^53^ Participants receiving RTSM indicated acceptable use of the intervention, but also reported frustration when it interfered with their daily lives.^53^

### Predictors of outcome

Four studies explored relevant predictors of treatment outcome, including demographic variables, autism diagnosis, co-occurring mental health difficulties and verbal IQ, but all showed no effect on change in outcomes (Supplementary Table 12).

## Discussion

The current systematic review aimed to identify strategies used to improve mental healthcare for autistic adults and examine their feasibility, acceptability and effectiveness. A total of 30 studies were included. A variety of approaches to adapted and bespoke mental health interventions and service improvements were identified.

Most studies reported on strategies that were adapted mental health interventions. These were largely CBT-based, targeting anxiety or a combination of mental health difficulties, with a few targeting depression and OCD. Adaptations to these interventions included mainly communication accommodations, modifications to their structure and content, and tailoring treatment to individual differences. We also identified service adaptations aimed to improve mental healthcare for autistic people and the identification of autism. Adaptations to services included communication accommodations, clinicians’ increasing knowledge and detection of autism, environmental adjustments and accommodating individual differences. Bespoke mental health interventions of CBT for social anxiety, CBT for anxiety in combination with virtual reality, AAT and RTSM were also identified.

Evidence on effectiveness of the strategies was generally of low quality and inconclusive, as most included studies lacked a comparison group, and RCTs were not sufficiently powered to detect significant differences between groups. Moreover, there were no trials comparing adapted and non-adapted mental health interventions; consequently, the extent to which adaptations were an improvement on standard mental health interventions could not be determined.

Overall, most bespoke and adapted mental health interventions appeared to be acceptable and feasible. Qualitative evidence from participants showed that adaptations such as visualisation^51^ in CBT for anxiety and the use of simple language and a concrete and structured approach in guided self-help CBT for depression^39^ were perceived as helpful. On the other hand, the pacing of sessions of guided self-help CBT for depression and the use of pre-defined visual tools received differing views.^39^ Therapists reported frequently using adaptations related to communication, content of the intervention and adjustment of treatment to individual needs when delivering CBT and EMDR to autistic people.^43,44^ However, one study with service users reported limited feasibility of adapted CBT for OCD, which could be attributed to difficulties attending sessions regularly.^52^ Another study demonstrated limited feasibility and acceptability of bespoke RTSM,^53^ as the unpredictability of the intervention led to frustration and interference with life.

Autism-tailored service pathways and models were found to be acceptable and feasible, suggesting that the introduction of autism-specific training could develop clinicians’ knowledge and skills and improve care. Initiatives to improve the identification of autism also indicated that the use of Autism-Spectrum Quotient, RAADS-R and ASD-DF to identify potentially autistic people could be helpful for screening for autism in mental health settings, and subsequent referral to specialist autism assessment.^41,42^ However, only two studies investigated the detection of autism, despite the perceived misdiagnosis of autism and the impact this may have on treatment.^16^

A variety of adaptations to services and interventions were identified that are acceptable and feasible to implement in mental healthcare. Many of these adaptations have been prioritised by autistic adults,^60^ including improving communication, providing environmental adjustments and developing clinicians’ knowledge of autism. This is a promising finding, as it demonstrates that autistic people's priorities are being taken into consideration. However, some adaptations (e.g. communication) were more frequently used than others (e.g. environmental adjustments, clinician knowledge, detection of autism), indicating that there may be scope for further improvement in service provision. Notably, we identified a lack of comprehensive descriptions of how interventions were adapted and why. Furthermore, no tailored pharmacological interventions or prescription initiatives were found, regardless of the common use of psychotropic medication,^61^ which is perceived as inappropriate by some autistic people,^11^ highlighting a potential gap. Other notable gaps include evidence on what works for autistic people with intellectual disabilities, suggesting a selection bias against participants with intellectual disabilities,^62^ and evidence on supporting autistic people with severe or long-term mental health difficulties including high levels of suicidality and self-harm. These are important areas in the context of the disproportionate detention of autistic people to psychiatric wards.^63^

The current review also piloted a novel and lived experience researcher-led Autism-Inclusive Research Assessment. Only 24% of studies involved autistic individuals, hence co-produced research is lacking. Selection of outcomes measures and data collection methods in many of the papers demonstrated a lack of adjustments to facilitate wider participation: some interventions appeared to focus on masking autistic traits, and language used showed a deficit approach to autism.^64,65^

### Strengths and limitations

The current review identified a list of adaptations that have been implemented across the service pathway, which may be helpful for tailoring treatments and services to the needs of autistic people. Another key strength is that the review was co-produced with lived experience researchers, who were involved in all aspects of the review, including protocol development, article screening, data extraction and synthesis, Autism-Inclusive Research Assessment, interpretation of findings, write-up and dissemination. We used the GRADE framework to assess quality of evidence for effectiveness and integrated it within our narrative synthesis.

Our review has several limitations. While we independently reviewed in duplicate the full text of all eligible studies, we did not double-screen all titles and abstracts. An additional limitation is that most studies included in the review had a small sample and lacked a comparison group, preventing us from attributing any improvements in outcomes to the intervention alone, and where there were comparisons, they were not with a non-adapted intervention, hindering any conclusions on effects of adaptations. A further limitation is that a high proportion of participants were White and male, neglecting underrepresented groups such as Black and minority ethnic and other gender identities. Differences associated with gender, ethnicities and cultures may be implicated in the presentation of autistic traits and may impact how adaptations are received by these groups, thus should be taken into consideration.^66–69^ Additionally, research on different types of strategies (e.g. adaptations to pharmacological interventions) and research including autistic people with intellectual disabilities and severe or long-term mental health difficulties were notably missing. The distinction between bespoke and adapted mental health interventions was also not always clear-cut, therefore these may not be particularly different, which may be because some interventions lacked a detailed description.

### Clinical implications and future directions

The current review highlighted a list of strategies evaluated as acceptable and simple to implement, such as improving communication and providing environmental adjustments, that do not necessarily require a further RCT evaluation. An individually tailored approach to treatment may be particularly helpful in facilitating appropriate mental healthcare, as autistic people differ in their support needs and presentation of autistic traits,^70,71^ and have also been shown to benefit less from standard evidence-based psychological therapy than adults without identified autism.^72^ Nevertheless, a balance should be struck between tailoring treatment to individual differences and adhering to evidence-based practice, which could possibly be addressed through a neurodivergence-informed approach to therapy and primary research.^26^

Future research should investigate a co-produced package of mental health service improvement measures. There is also a need for recruitment strategies that increase participation from underrepresented groups and reduce biases (e.g. male bias); and for an increased focus on those with intellectual disabilities and/or severe or long-term mental health difficulties.

## Conclusion

This co-produced systematic review with autistic people and carers of autistic people identified strategies that have been used to improve mental healthcare for autistic individuals, of which most were adapted CBT-based mental health interventions addressing anxiety. The developed list of adaptations may help tailor treatments and services to the needs of autistic people. The evidence on effectiveness remained inconclusive because of methodological limitations in many of the included studies, whereas the evidence for feasibility and acceptability was largely positive. Additionally, many of the identified adaptations to services and interventions are simple, reasonable adjustments that align with general good clinical practices and may not require further research. The clinical implications highlight the importance of tailored approaches incorporating adaptations, such as adapting the style of communication and creating a more autism-friendly environment, offering actionable insights for clinicians. The review's findings suggest that more robust research is needed, and future research should prioritise a co-produced package of mental health service improvement measures, incorporate a neurodivergence-informed approach to therapy and address biases in recruitment strategies to enhance mental healthcare for autistic adults.

### Lived experience commentary, written by Suzi Sapiets and Amanda Timmerman

This review identified several adapted approaches to mental health support for autistic adults which appeared to be feasible and acceptable, such as increasing knowledge and identification of autism, environmental adjustments, accommodations for communication and individual differences, and modifications to the structure and content of interventions. However, because of a lack of statistical power and comparison groups, there was no conclusive evidence on effectiveness.

As autistic adults with experience of mental health services, it is encouraging to see that adaptations have been implemented to improve support for autistic people. However, it is important to acknowledge that accessing support is not straightforward for autistic adults because of various barriers (e.g. professionals lacking appropriate knowledge of autism, lack of preferred communication methods, stigma, complex referral pathways, long waiting lists, inappropriate and short-term therapies, lack of trauma focus, cost). This is problematic considering UK services have a legal duty to make reasonable adjustments under the Equality Act 2010.

Nonetheless, many of the strategies are small and easy to do (e.g. lighting adaptations, gender considerations, adjusting language/communication). Therefore, mental health services could implement small changes to improve support for autistic people. Allowing autistic people to communicate their needs in advance of therapy sessions may aid this process, particularly when clients are anxious. Furthermore, therapists could make special considerations for people recently diagnosed with autism or who have limited experience of addressing their accessibility needs, by recommending potential adjustments.

The review also found strategies to improve the identification of autism in mental health services. This finding is critical, as services likely support autistic people who do not have an autism diagnosis. Intersectionality is important, as many people face disproportionate barriers to diagnosis and support (e.g. gender, ethnicity, culture).

The studies identified in this review did not appear to include the perspectives of autistic therapists who support autistic clients. Receiving support from an autistic therapist can be transformative, as one of us has experienced. Ongoing adaptations, shared understanding of neurodivergence, similar communication styles and the therapist's specialism in trauma resulted in exceptional support. Future research could explore the potential benefits of autistic therapists supporting autistic clients.

Overall, this review highlighted key areas in need of attention when providing mental health support for autistic people. We hope these findings will increase the likelihood that autistic people will receive more appropriate support, and potentially benefit other neurodivergent people seeking support also.

## Supporting information

Loizou et al. supplementary material Loizou et al. supplementary material

## Data Availability

Data were collected from publicly available research papers which are referenced.

## Footnotes

‡ Although there are different terms used to refer to people on the autism spectrum, in this review we are using identity-first language (e.g. ‘autistic person’)
